# Overshadowing as prevention of anticipatory nausea and vomiting in pediatric cancer patients: study protocol for a randomized controlled trial

**DOI:** 10.1186/1745-6215-14-103

**Published:** 2013-04-20

**Authors:** Friedemann Geiger, Levke Wolfgram

**Affiliations:** 1 Department of Pediatrics, University Medical Center Schleswig-Holstein, Campus Kiel, Schwanenweg 20, Kiel, 24105, Germany; 2 Tumor Center, University Medical Center Schleswig-Holstein, Campus Kiel, Kiel, 24105, Germany

## Abstract

**Background:**

Emesis and nausea are side effects induced by chemotherapy. These effects lead to enormous stress and strain on cancer patients. Further consequences may include restrictions in quality of life, cachexia or therapy avoidance. Evidence suggests that cancer patients develop the side effects of nausea and vomiting in anticipation of chemotherapy. Contextual cues such as smell, sounds or even the sight of the clinic may evoke anticipatory nausea and vomiting prior to infusion. Anticipatory nausea and vomiting are problems that cannot be solved by administration of antiemetica alone.

The purpose of the proposed randomized placebo-controlled trial is to use an overshadowing technique to prevent anticipatory nausea and vomiting and to decrease the intensity and duration of post-treatment nausea and vomiting. Furthermore, the effect on anxiety, adherence and quality of life will be evaluated.

**Methods/Design:**

Fifty-two pediatric cancer patients will be evenly assigned to two groups: an experimental group and a control group. The participants, hospital staff and data analysts will be kept blinded towards group allocation. The experimental group will receive during three chemotherapy cycles a salient piece of candy prior to every infusion, whereas the control group will receive flavorless placebo tablets.

**Discussion:**

If an effectiveness of the overshadowing technique is proven, implementation of this treatment into the hospitals’ daily routine will follow. The use of this efficient and economic procedure should aid a reduced need for antiemetics.

**Trial registration:**

Current Controlled Trials ISRCTN30242271/

## Background

Around 1,800 children (under 15 years old) per year develop cancer in Germany [1]. The chances of surviving childhood cancer have increased considerably in the past 30 years due to differentiated diagnostic and developments in the therapy regimes. Today 83% of all pediatric cancer patients survive the first 5 years after diagnosis, an increase from 67% in the 1980s. A reasonable proportion of this development can be attributed to progress in cytostatics. However, the well-known side effects have remained. The typical side effects are nausea and vomiting. Experiences of nausea and vomiting can lead to anxiety, restrictions in quality of life and reduced adherence to therapy. In the proposed randomized controlled trial the effectiveness of an intervention technique called overshadowing on chemotherapy-related nausea and vomiting will be investigated. Furthermore, the impact on anxiety, adherence and quality of life will be studied. The following sections describe the conceptualities.

### Emetogenity of cytostatics

Aside from the desired effect of tumor reduction, cytostatics affect a number of organ systems. Amongst other systems, cytostatics stimulate the area postrema, a circumventricular organ that lies outside the blood–brain barrier, stimulation of which can lead to vomiting [2]. Nausea and vomiting are considered by patients to be the most burdening adverse reactions and the most abundant reasons to terminate therapy. Physiologically, nausea and vomiting raise the risk of developing Mallory–Weiss syndrome. Furthermore, prolonged nausea and vomiting could produce exsiccosis, cause electrolyte imbalance and lead to a high level of weight loss [3].

The frequency of chemotherapy-induced emesis depends primarily on the emetogenic potential of the cytostatics. The Multinational Association of Supportive Care in Cancer (MASCC) classifies the cytostatics in four emetic risk groups. A high-level agent produces emesis in nearly all patients (>90%), a moderate-level risk in 30 to 90% of patients, a low-level risk in 10 to 30% of patients and the minimal level tends to show risk in <10% of patients [4]. Table 1 presents the emetic risk groups of cytostatics.

**Table 1:** Emetic risk groups according to MASCC

**Risk**	**Cytostatics**
High	Cisplatin, mechlorethamine, streptozocin, cyclophosphamide >1,500 mg/m^2^, carmustine, dacarbazin
	*Oral*: Hexamethylmelamine, procarbazine
Moderate	Oxaliplatin, cytarabine >1,000 mg/m^2^, carboplatin, ifosfamide, cyclophosphamide <1,500 mg/m^2^, azacitidine, alemtuzumab, doxorubicin, daunorubicin, epirubicin, idarubicin, irinotecan, bendamustine, clofarabine
	*Oral:* Cyclophosphamide, temozolomide, vinorelbine, imatinib
Low	Paclitaxel, docetaxel, mitoxantrone, topotecan, etoposide, pemetrexed, methotrexate, doxorubicin HCl liposome injection, temsirolimus, ixabepilone, mitomycin, gemcitabine, cytarabine <1,000 mg/m^2^, 5-fluorouracil, bortezomib, cetuximab, trastuzumab, catumaxomab, panitumumab
	*Oral:* Capecitabine, tegafur uracil, etoposide, sunitinib, fludarabine, everolimus, lapatinib, lenalidomide, thalidomide
Minimal	Bleomycin, busulfan, cladribine, fludarabine, vinblastine, vincristine
	vinorelbine, bevacizumab
	*Oral*: chlorambucil, hydroxyurea, melphalan, methotrexate, 6-thioguanine
	gefitinib, sorafenib, erlotinib

### Post-treatment nausea and emesis

Chemotherapy-induced nausea and/or emesis are commonly classified as acute, delayed, anticipatory, breakthrough or refractory [5]. Acute onset usually occurs within a few minutes to 1 or 2 hours after infusion and resolves within the first 24 hours. Delayed onset emesis begins or persists more than 24 hours after chemotherapy treatment. Anticipatory nausea and emesis occurs before patients receive their chemotherapy administration. Breakthrough emesis occurs despite prophylactic treatment and requires rescue antiemetics. Refractory emesis arises during subsequent treatment cycles when antiemetic prophylaxis and rescues have failed in earlier cycles.

Chemotherapy-induced nausea and vomiting differs from that usually experienced – it lasts longer, its degree of severity varies from treatment to treatment and there is a greater variability in patient reaction. For example, anxiety, personality and environment seem to play a key role. Factors that increase the risk of nausea and emesis beside pharmacological (dosage, agent, duration) are age, gender and expectation of these adverse effects [6].

Initiation and coordination of the emetic process is the responsibility of the vomiting center, a structure located in the lateral reticular formation of the medulla. Afferent input from several sources, including the higher brain stem and cortical structures, are capable of initiating the emetic process [7].

### Antiemetics

The MASCC published guidelines for the use of antiemetics [4]. For adult patients with high emetic risk from chemotherapy, a combination of a 5-HT3 receptor antagonist, dexamethasone, and aprepitant is recommended prior to chemotherapy.

For patients who receive moderate emetic-risk chemotherapy, not including a combination of anthracycline plus cyclophosphamide, palonosetron plus dexamethasone is recommended for prophylaxis of acute nausea and vomiting. Patients who receive moderately emetic chemotherapy known to be associated with a significant incidence of delayed nausea and vomiting should receive antiemetic prophylaxis for delayed emesis. A single antiemetic agent such as dexamethasone, a 5-HT3 receptor antagonist, or a dopamine receptor antagonist, such as metoclopramide, is suggested for prophylaxis in patients receiving agents of low emetic risk. No antiemetic should be administered for the prevention of delayed emesis induced by low or minimally emetic chemotherapy. The MASCC describes that the best approach for anticipatory emesis is the best possible control of acute and delayed emesis. The guidelines for the chemotherapy-induced prevention of nausea and vomiting for high and moderate risk in children states that all patients should receive antiemetic prophylaxis with a combination of a 5-HT3 receptor antagonist and dexamethasone. There are currently no appropriate studies available for the prevention of delayed anticipatory nausea and vomiting (ANV) or for the prevention of nausea and vomiting following chemotherapy of minimal and low emetic risk in children. No formal recommendation is thus available. The MASCC suggests that children should be treated in a manner similar to that of adults receiving chemotherapy with appropriate doses. This limited level of standardization may lead to widely varying antiemetic strategies in different centers. However, the MASCC recommendations are similar to, for example, those given in the protocol for one of the largest therapy-optimizing studies worldwide [8], for acute lymphoblastic leukemia.

Ihbe-Heffinger and colleagues observed that the majority of their adult patients (64.4%) experienced nausea and emesis, although they took prophylactic medication [9]. More patients experienced delayed than acute nausea and emesis (60.7% vs. 32.8%), and more patients reported nausea than vomiting (62.5% vs. 26%). The authors concluded that antiemetic medications could control acute rather than delay emesis and should effect a reduction in the frequency of vomiting but not in episodes of nausea.

### Anticipatory nausea and emesis

As already mentioned, many cancer patients not only experience the side effects of nausea and emesis after chemotherapeutic drug infusion, but also prior to treatment [10]. These symptoms are known as ANV. The incidence ranges from 18 to 57% and nausea is more common than vomiting [5]. The reported rates vary widely among studies. Morrow and colleagues found in their meta-analysis of 35 studies an average prevalence of 29% for adult and pediatric patients [11]. Despite modern antiemetic treatment, ANV still occurs in 25 to 30% of cases [12].

The etiology of ANV can be explained by classical conditioning established by Pavlov (1849 to 1936). During conditioning an organism learns to associate an initial neutral stimulus (the conditioned stimulus) with a biologically relevant stimulus (the unconditioned stimulus). By pairing a conditioned stimulus with an unconditioned stimulus in the acquisition phase, the conditioned stimulus comes to evoke a conditioned response that is commonly similar to the response elicited by the unconditioned stimulus [13].

Accordingly, contextual stimuli of the clinic environment, such as the smell, sounds and sight of the building, function as the conditioned stimulus that becomes associated with the unconditioned stimulus of chemotherapy treatment. Following one or more contingent pairings (chemotherapy infusions), the patient may develop the conditioned response of nausea and/or vomiting even before the next treatment just by seeing the infusion, meeting the same clinician or already while re-entering the clinic [14].

As shown by Hickok and colleagues, the development of ANV coheres with the emetogenicity of the chemotherapy drug [15]. Beyond that, Tyc and colleagues showed that occurrence of ANV is positively correlated with severity of vomiting (intensity, frequency, duration) and number of chemotherapy cycles (conditioning trials) [16]. ANV is further inversely correlated with patient age, according to Morrow [17].

ANV is also seen in animal models. Limebeer and colleagues observe that, although rats do not vomit, they display a distinctive gaping reaction when exposed to a toxin-paired flavored solution [14]. After several pairings the contextual cues elicit a conditioned state of nausea in rats.

### Quality of life

Quality of life is defined as a health-related multidimensional construct that includes physical, emotional, mental, social and behavioral components of well-being and functioning from the viewpoint of patients respective to observers [18].

Calaminus and colleagues found that patients who survived childhood cancer estimate their quality of life to be as good as that of healthy children of the same age [19]. However, the various aspects of quality of life are judged differently among the diverse oncological domains. For example, children with solid tumors show less impairment than children with leukemia; one could therefore suggest that a diagnosis at young age and a longer period of being dependent on family support, isolation from peer groups and delayed independence may be reflected by this result [19].

Previous studies estimate an influence of nausea and emesis on cancer patients’ quality of life [20,21].

As shown by Akechi and colleagues, the presence of anticipatory nausea was significantly affecting most domains of patients’ quality of life [22]. This influence maintains when controlling for age, sex, performance status, and psychological distress.

### Anxiety

State anxiety (as opposed to trait anxiety) is defined as an emotional process signed through arousal, worries, nervousness, inner restlessness and fear of future events. State anxiety varies in intensity, time and situation [23]. Anxiety is the result of threats that are perceived to be uncontrollable or unavoidable [24].

State anxiety is associated with incidence and severity of post-treatment vomiting, and varies inversely with the emetic potential of the chemotherapy regimen [7]. This counterintuitive finding might be explained by psychological factors being relevant in the experience of post-treatment vomiting for regimens of low to moderate emetic potential while their impact might be reduced or minimal for regimens with high emetic potential.

State anxiety might foster development of ANV as it facilitates classical conditioning of anticipatory responses [17]. A review by Andrykowski comprising 12 studies showed mixed results [25]. The relationship between anxiety and ANV seems unclear.

A study with pediatric cancer patients found no significant differences in state anxiety scores between patients, whether or not they experience ANV [26].

### Compliance/adherence

Compliance was previously defined as the willingness to follow medical advice. Understanding of the patient’s role, however, has changed in recent decades. As a consequence the term adherence is increasingly used instead of compliance. Adherence expresses an active patient role with the aim to create a cooperation based on agreement between physicians and patients that should lead to maintenance in therapy regimens [27]. In the literature, compliance is still synonymously used for adherence. Reasons for nonadherent behavior are shown in Table 2.

**Table 2:** **Reasons for nonadherent behavior of patients **[45]

	
Patient-related factors	Therapy-related factors
• Low level of suffering	• Complexity of regimen
• Anxiety that therapy harms	• Side effects
• Anxiety that medication leads to nausea/vomiting, and so forth	• Long-term therapy
• Syringe phobia	
• Negative experiences related to clinic or doctor	
• Laziness, restrictions in intelligence or memory	
Doctor–patient relationship	Institution
• Insufficient education and information	• Longer waiting times
• Dissatisfaction	• Insufficiently structured processes
• Long consultation interval	

Predictors for adherent behavior from pediatric cancer patients mentioned in the review by Tebbi are the mode of application, satisfaction with medical supply, inner belief of control and age [28]. Adolescents often showed nonadherent behavior. Factors such as gender, parental income or family status had no influence on adherence.

Adherence is important for treatment success. A low degree of adherence is found to lead to increased mortality [27].

### Overshadowing

The phenomenon of overshadowing was first observed by Pavlov [29]. When two or more stimuli are present, the more salient one produces a stronger response than the other. The presence of the more salient element is commonly found to restrict the acquisition of associative strength by the less salient element [30].

Pavlov explained it as follows: ‘The effect of the compound stimulus is found nearly always to be equal to that of the stronger component used singly, the weaker stimulus appearing therefore to be completely overshadowed by the stronger one’ [29].

Transferring the overshadowing paradigm to chemotherapy processes, a salient stimulus presented during drug infusion may overshadow the effects of the less salient one (the doctor’s white coat). The conditioned response elicited by the less salient stimuli is weakened through the overshadowing element. This weakening prevents the development of ANV [10]. According to Garcia and Koelling, tastes become more associated with stimuli causing nausea and vomiting [31]; they are more salient than other sensational perceived stimuli.

Examination of the psychological, medical and nursing literature in PubMed for the overshadowing procedure, also known as the scapegoat technique from classical conditioning, revealed a total number of 124 findings (see search strategy in Table 3). The majority of studies attend to foundational research of overshadowing; for example, the involved brain functions during associative learning.

**Table 3:** Search terms and results of the systematic literature review

**Number**	**Queries**	**Result**
#14	Search (#7) AND #3 Limits: Randomized Controlled Trial, Cancer	0
#13	Search (#7) AND #3 Limits: Cancer, Young Adult: 19–24 years	0
#12	Search (#7) AND #3 Limits: Cancer, All Child: 0–18 years	0
#11	Search (#7) AND #3 Limits: Cancer	2
#10	Search (#7) AND #3	4
#9	Search (#1) AND #2 Limits: Cancer, All Child: 0–18 years	1
#8	Search (#1) AND #2 Limits: Cancer	6
#7	Search (#1) AND #2	124
#6	Search #4 Limits: Humans, Cancer, All Child: 0–18 years	2
#5	Search #4 Limits: Humans, Cancer	33
#4	Search #1 Limits: Humans	263
#3	Search ((((((anticipatory nausea) OR anticipatory emesis) OR anticipatory vomiting) OR chemotherapy-related nausea) OR chemotherapy-related vomiting)) OR chemotherapy-related emesis	531
#2	Search (((classical conditioning) OR pavlovian conditioning) OR pawlowian conditioning) OR conditioned	84,626
#1	Search (overshadowing) OR scapegoat	463

A limited search focusing on cancer leads to four articles about the scapegoat effect on food aversion from cancer patients [32-35], while two articles consider the overshadowing effect on conditioned nausea [10,36]. Of these, just one describes an investigation among pediatric cancer patients [34]. Screening of the reference lists of these articles did not add any previously unconsidered publications.

Broberg and Bernstein used a scapegoat technique to prevent food aversion in children undergoing chemotherapy [34]. Patients received candy (coconut and root beer Lifesavers) between the consumption of a meal and administration of chemotherapy. Children who received the candy, which served as a scapegoat, were twice as likely to eat some portion of a future test meal.

Stockhorst and colleagues investigated 16 adult cancer patients with an overshadowing protocol using salient drinks to prevent anticipatory nausea and emesis [10]. The experimental group (*n* = 8) received salient drinks before administration of drug infusions through two cycles of chemotherapy, while the control group received water. In the third cycle of chemotherapy all patients received water. Patients receiving an overshadowing treatment did not develop anticipatory nausea, whereas two patients of the control group did. Furthermore, overshadowing tended to modify the occurrence of post-treatment nausea: it occurred later and was of shorter duration. The results are not statistically significant because of a quite small sample size; they only suggest tendencies.

In a pilot study at our medical center, Görges adapted the study design of Stockhorst and colleagues [10] for the pediatric setting (*n* = 30), where overshadowing proved to be effective [37]. No patient of the overshadowing group (*n* = 15) developed anticipatory nausea, compared with 13 patients of the control group. Furthermore, overshadowing reduced the occurrence of concomitant symptoms such as anxiety, nonadherent behavior and affected well-being. Overshadowing also seemed to decrease the intensity of post-treatment nausea. However, the partially insufficient feasibility of the used intervention technique led to problems of recruitment. This problem may have biased the results towards an overestimation of the intervention effect. Such threats of validity have to be avoided in the present study. Additionally, reducing the complexity of the intervention increases the chances of implementation into daily clinical routine.

### Aims of the study

The aims of the present study were to verify the effect of an optimized overshadowing technique on ANV (primary endpoints), and to further investigate the intervention effect on post-treatment nausea and vomiting (secondary endpoints). The subgoals were to investigate the overshadowing effect on patients’ quality of life, state anxiety and adherence; to survey the relation between prevalence of post-treatment and ANV; and to determine the applicability of the overshadowing treatment in the hospital’s daily routine.

## Methods/Design

### Ethical approval

The study protocol was approved by the ethics committee of the Christian-Albrechts-University, Medical Faculty, Kiel, Germany (6 March 2012, reference number A 168/11).

Participants and their parents shall receive written information and are required to give their written consent prior to participation.

### Participants

Newly diagnos ed pediatric patients with an oncological disease shall be included in the Kiel University clinic. These children, adolescents and young adults must also meet further inclusion criteria: German speaking, over the age of 4 years, and receiving chemotherapy. To guarantee a proper acquisition phase of overshadowing, the children should run through at least three chemotherapy cycles. An interval of 7 days between each chemotherapy cycle is important to differentiate between post-treatment and ANV. Children with brain or gastrointestinal tract cancer will be excluded to eliminate an organic cause of nausea and vomiting. Other exclusion criteria include mental restrictions, recurrent cancer, and received radiotherapy. Finally, pediatric patients should not have experienced treatment-related nausea or vomiting before.

### Sample size calculation

The calculation of the required sample size is oriented towards the demands of the first study question, which addresses the effect of overshadowing on ANV (primary endpoints). The effect sizes that were achieved in our pilot study for reduction of ANV are to be judged as very large in view of statistical conventions (effect sizes in *t* tests were *d* = 2.5 (nausea) and *d* = 1.4 (vomiting)). For the present study, a more conservative effect size of *f* = 0.4 in analysis of covariance (see below) is assumed. Aiming at a statistical power of 0.80, a total sample size of *n* = 52 is needed (two-sided α = 0.05).

### Study design

**Figure 1:**
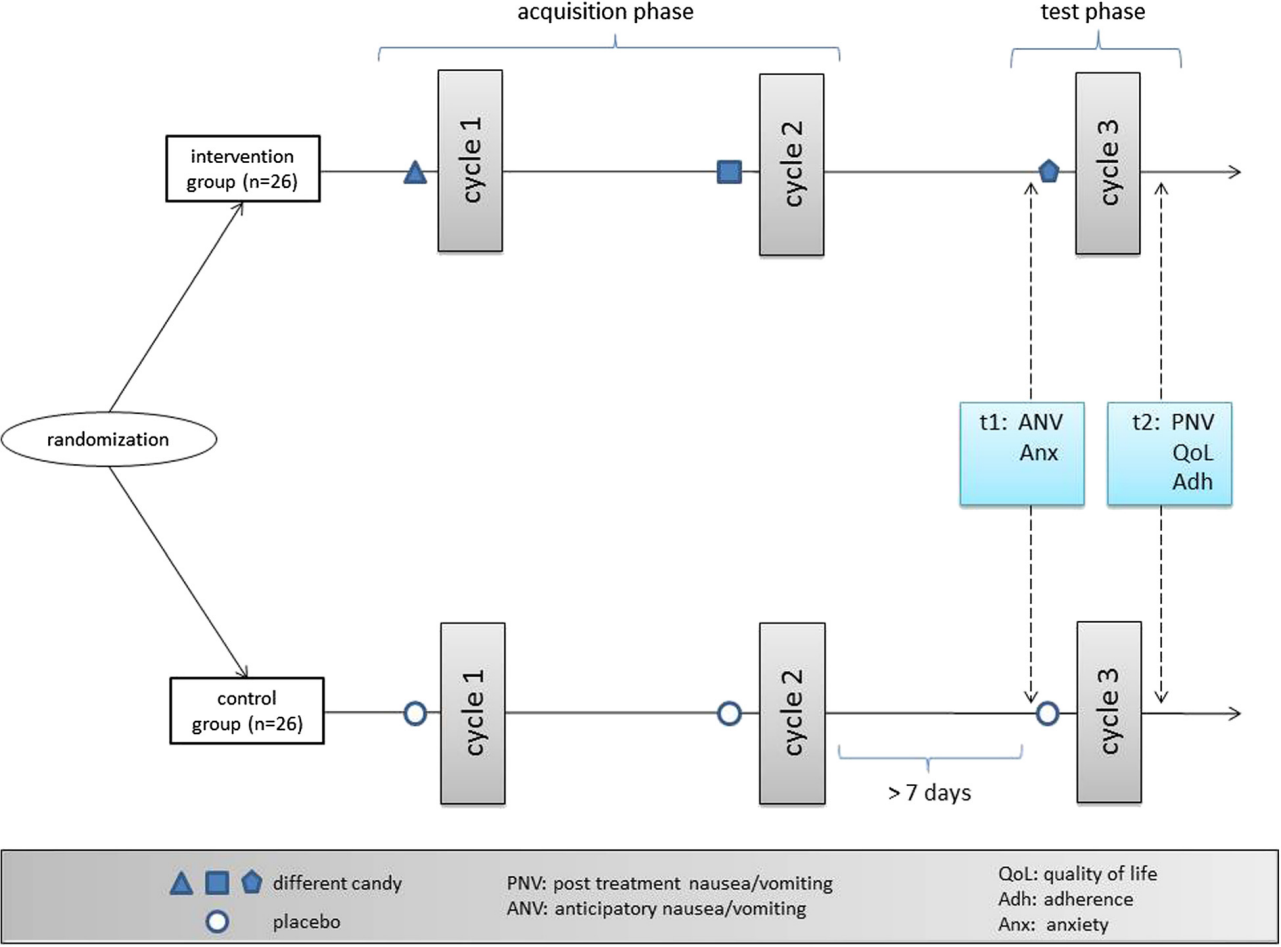
Study design and flow of participants.

The study is a monocenter randomized controlled trial comparing groups of pediatric cancer patients undergoing an overshadowing treatment during chemotherapy or receiving a placebo treatment. Figure 1 shows the study design.

Different diagnoses require different cytostatics. Drugs, in turn, influence the probability of usage and the type of antiemetics. Block randomization will therefore be conducted within each diagnosis (that is, within the group of patients suffering from acute lymphoblastic leukemia or those suffering from Ewing’s sarcoma, and so forth). According to the recommendations of Altman and Bland [38], separate block randomization lists for each stratum will be used. This process is carried out by an independent third person not involved in data collection, analysis or medical care.

Participants, medical staff and data analysts will be kept blinded towards allocation. While blinding of medical staff and data analysts is easily realized, participants may notice if they get candy with a salient taste (experimental group) or without (control group). However, as the study information leaflets do not refer to taste in particular but to the effect of melting something in one’s mouth, they are not able to conclude their group allocation by their intervention. Accordingly, participants can also be considered blinded.

### Intervention

In the mentioned pilot study the overshadowing treatment known from the paradigm of classical conditioning was tested on pediatric cancer patients. To increase efficiency and applicability, an overshadowing technique known from Broberg and Bernstein will be used [34]. Implementing overshadowing in the hospital’s daily routine requires easy handling and regard to the high hygienic standards on the oncology ward, although in the proposed study the experimental group will receive salient candy and the control group flavorless placebo tablets instead of drinks. During three chemotherapy cycles the experimental group will get their treatment (candy) prior to each infusion. To avoid influences of possible aversive taste reactions, the flavor of candy will be changed from infusion to infusion.

Determining an evaluation period of three cycles can be seen as a compromise. On the one hand, more cycles may increase the intervention effect compared with the placebo condition. On the other hand, more cycles also increase the danger of withdrawals, missing data and comparability of participants and their treatment as the total number of treatment cycles differs widely between low-risk Hodgkin lymphoma and Ewing sarcoma, for example. Three cycles are thus a trade-off between a large effect size and threatened data quality. The pilot study [37] and the basic research [39] showed that even less than three cycles – where each comprises several unconditioned stimulus–conditioned stimulus pairings – is sufficient. However, to determine the applicability of the intervention in daily clinical routine (the third subgoal), the overshadowing treatment in the experimental group will be continued until the end of treatment without any further outcome assessment whenever possible.

### Measures

#### Measuring nausea and vomiting

Symptoms of nausea and emesis will be measured using discomfort logs administered by patients. These logs have been successfully used in our pilot study [37]. They assess nausea and vomiting on 6-point Likert scales focusing on intervals of 2 hours, thus leading to 12 ratings every day. Children from 4 to 7 years of age will be interviewed by a blinded interviewer about their symptoms, and also parents will be asked about their observations. Children from the age of 8 years will use a self-assessment version.

As a second measure, the combined nausea and vomiting scale Baxter Retching Faces (BARF) by Baxter and colleagues will be used [40]. BARF uses a 6-point visual analogue scale with six comic faces expressing mimics ranging from neutral mood to vomiting. The authors developed the BARF scale for children aged from 4 to 17 years. The validation study confirms reliability and validity among patients from 7 to 18 years old. In our study, the BARF scale will be administered by all patients.

There will be two measurement points: the anticipatory measurement phase prior to the third chemotherapy cycle, and also the post-treatment phase after the third chemotherapy cycle (see Figure 1 and Table 4).

**Table 4:** Planned measurements for study aims and subgoals

**Measurement**	**Pre-chemotherapy cycle 3 (anticipatory), t1**	**Post-chemotherapy cycle 3, t2**
Nausea and vomiting (discomfort log, BARF)	X	X
Anxiety (KAT-II, STAI)	X	
Quality of life (Kiddy-KINDL, Kid-KINDL, Kiddo-KINDL)		X
Adherence (caregivers and physicians questionnaires)		X

*BARF*, Baxter Retching Faces; *KAT-II*, Kinder-Angst-Test (Children Anxiety Test); *KINDL*, German Children's Quality of Life Questionnaire (Kiddy/Kid/Kiddo version for each age group); *STAI*, State and Trait Anxiety Inventory.

#### Measuring anxiety

The Kinder-Angst-Test-II/Children Anxiety Test-II [41] is a revision of the questionnaire for German-speaking children and adolescents referring to the trait concept developed in 1969. The trait scale for measuring the anxiety disposition was kept and extended to aspects of state anxiety and was normalized. The revised Kinder-Angst-Test-II consists of three questionnaires, acquiring two different aspects of anxiety. One questionnaire (Form A) appraises trait anxiety, whereas the two others (Form P, Form R) appraise the state anxiety, particularly the anticipated respectively reminded anxiety. The age range for application reaches from 9 to 16 years.

Form A consists of 20 items. The questionnaires Form P and Form R consist of 12 items each, with differences to the referred time period. To operationalize state anxiety, questionnaire Form P will be used prior to the third chemotherapy cycle (see Table 4).

The State and Trait Anxiety Inventory [42] is a self-report inventory with 20 items each for trait and state anxiety (two questionnaires). Items are rated on a four-level scale, ranging from not at all to very, to measure the intensity of anxiety. Adolescents older than 15 years will be only asked to complete the state anxiety questionnaire. The state scale will be assessed prior to the third chemotherapy cycle (see Table 4).

#### Measuring quality of life

The German Children's Quality of Life Questionnaire (KINDL) – Revised is a quality-of-life questionnaire for children with 24 items and allows assessments of six domains: physical well-being, psychological well-being, self-esteem, family, friends, and daily function [43]. Reliability scores (Cronbach’s α = 0.85) and validity of the instrument are confirmed [44]. There are three different self-assessment versions and two parents’ versions: Kiddy-KINDL interview for children aged from 4 to 7 years of age, Kid-KINDL for children aged from 8 to 12, Kiddo-KINDL for adolescents aged from 13 to 16, Kiddy-KINDL parent’s version for children aged from 4 to 7, and KINDL parent’s version for children and adolescents aged from 8 to 16 years of age.

To assess quality of life, pediatric patients will complete the KINDL-R versions after the third chemotherapy cycle (see Table 4).

#### Measuring adherence

Adherent behavior will be obtained through ratings from caregivers and physicians. As in the primary study, the caregivers and physicians administer a questionnaire with eight items to assess the adherent behavior of the children and adolescents. Answers – for example, the intake of medicine – can be rated on a four-level scale from poor to very good (poor, a little, good, very good). The rating of patients’ adherent behavior will proceed after the third chemotherapy cycle (see Table 4).

### Statistical analysis

The effect of the overshadowing treatment on both anticipatory (t1) and post-treatment nausea and vomiting, anxiety, quality of life and adherence (t2) will be analyzed using analysis of covariance comparing mean scores of intervention and control group while controlling for dosage of antiemetics. To account for different appropriate absolute dosages (mg/kg or mg/m^2^) among different patients and drugs, the variable dosage will be operationalized as the percentage of the recommended maximum dosage for each patient and drug. The assumed dependence between the prevalence of post-treatment (t2) and ANV (t1) will be determined by Pearson correlation coefficient.

## Discussion

### Timetable

In consideration of the number of patients and the distribution of diagnoses in previous years, 23 patients per year in the study clinic would meet the inclusion criteria. Extrapolating this number leads to an inclusion period of approximately 2.5 years. If the number of participants is less than expected, the investigation will be extended to include a second center (a verbal commitment from University Clinic Lübeck was received). In that case the randomization procedure will be repeated in the second center. The trial is intended to start in summer 2013.

### Limitations

Several limitations of this study have to be mentioned. First, it is unclear whether it will be possible to produce balanced study arms regarding diagnoses and antiemetics, respectively. In cases of clear imbalances, their impact on the result has to be discussed. Second, it is an open question whether overshadowing will work equally independently of age. Additionally, it is unclear whether ratings of ANV are influenced by the administering person (self-rating vs. third-person rating). However, our pilot study did not reveal any influence of age or application mode. Third, a paradox conditioning might occur: since nausea and vomiting are expected to occur closely after the application of candy, the latter might be associated with nausea and vomiting. Hence, both might occur as a consequence of other candy independently of treatment situations. However, this will not influence the desired effect on ANV as t1 lies before application of candy in the context of the third treatment cycle. Unintended induction of nausea and vomiting in patients’ everyday life is seen as unlikely since the tastes used as scapegoats are rather rare.

## Trial status

Ready to start recruitment.

## Abbreviations

ANV: Anticipatory nausea and vomiting; BARF: Baxter Retching Faces; KINDL: German Children's Quality of Life Questionnaire; MASCC: Multinational Association of Supportive Care in Cancer.

## Competing interests

The authors declare that they have no competing interests. None of the authors received external funding for conceptualization of this study protocol.

## Authors’ contributions

FG and LW designed the study and statistical analysis together. Both authors read and approved the final manuscript. Accordingly, both authors share first authorship.
